# Evaluation of temporal surveillance system sensitivity and freedom from bovine viral diarrhea in Danish dairy herds using scenario tree modelling

**DOI:** 10.1186/s12917-016-0744-2

**Published:** 2016-06-21

**Authors:** Alessandro Foddai, Anders Stockmarr, Anette Boklund

**Affiliations:** Section of Epidemiology, National Veterinary Institute, Technical University of Denmark, Bülowsvej 27, DK-1870 Frederiksberg C, Denmark; Statistics and Data Analysis Section, Department of Applied Mathematics and Computer Science, Technical University of Denmark, Matematiktorvet, DK-2800 Lyngby, Denmark

**Keywords:** Temporal surveillance system sensitivity, BVD, Scenario tree, Freedom from disease

## Abstract

**Background:**

The temporal sensitivity of the surveillance system (TemSSe) for Bovine Viral Diarrhea (BVD) in Danish dairy herds was evaluated. Currently, the Danish antibody blocking ELISA is used to test quarterly bulk tank milk (BTM). To optimize the surveillance system as an early warning system, we considered the possibility of using the SVANOVIR ELISA, as this test has been shown to detect BVD-positive herds earlier than the blocking ELISA in BTM tests.

Information from data (2010) and outputs from two published stochastic models were fed into a stochastic scenario tree to estimate the TemSSe. For that purpose we considered: the risk of BVD introduction into the dairy population, the ELISA used and the high risk period (HRP) from BVD introduction to testing (at 90 or 365 days). The effect of introducing one persistently infected (PI) calf or one transiently infected (TI) milking cow into 1 (or 8) dairy herd(s) was investigated. Additionally we estimated the confidence in low (PLow) herd prevalence (<8/4109 infected herds) and the confidence in complete freedom (PFree) from BVD (< 1/4109).

**Results:**

The TemSSe, the PLow, and the PFree were higher, when tests were performed 365 days after BVD introduction, than after 90 days. Estimates were usually higher for the SVANOVIR than for the blocking ELISA, and when a PI rather than a TI was introduced into the herd(s). For instance, with the current system, the median TemSSe was 64.5 %, 90 days after a PI calf was introduced into eight dairy herds. The related median PLow was 72.5 %. When a PI calf was introduced into one herd the median TemSSe was 12.1 %, while the related PFree was 51.6 %. With the SVANOVIR ELISA these estimates were 99.0 %; 98.9 %, 43.7 % and 62.4 %, respectively.

**Conclusions:**

The replacement of the blocking ELISA with the SVANOVIR could increase the TemSSe, the PLow and PFree remarkably. Those results could be used to optimize the Danish BVD surveillance system. Furthermore, the approach proposed in this study, for including the effect of the HRP within the scenario tree methodology, could be applied to optimize early warning surveillance systems of different animal diseases.

## Background

In Denmark, Bovine Viral Diarrhea (BVD) is considered an exotic disease [1]. An eradication program was initiated in 1994 [2, 3] and in the period from 2007–2011, only 3 out of approximately 4000 dairy herds were diagnosed with BVD by bulk tank milk (BTM) testing. All dairy herds were screened quarterly by BTM, while beef herds were screened at abattoir by blood sampling. If the BTM is classified as positive, individual animals are tested to find at least one antibody positive (sample size determined to have 95 % herd sensitivity, assuming 10 % within-herd prevalence). If the positive herd status is confirmed, all non-antibody positive animals are tested to detect the viremic animals and persistently infected (PI) cattle are eliminated [1].

The BVD virus (BVDV) can cause uterine infections in pregnant cows, abortions, stillbirths or weak calves [4–6]. Cows exposed to BVDV in the first 120 days of pregnancy, can give birth to calves, which become PI [4, 5]. PI cattle shed the virus in large amounts throughout their lives, while other transiently infected (TI) animals shed the virus in small amounts for 2–3 weeks and become lifelong immune [4–6].

Because the Danish dairy herd size has increased remarkably since the eradication program started [1], an evaluation and eventual optimization of the Danish BVD surveillance system was considered necessary by the Danish Cattle Federation. An optimal early-warning system based on BTM testing should detect newly infected herds as soon as possible. Early-warning surveillance systems are those aimed to detect the “unexpected threats” in a timely way [7]. Hence an early-warning system should be based on a) the risk that some herds become infected and b) the time needed to detect antibodies in BTM. The latter is known to depend on the herd size and on the threshold prevalence of antibody positive milking cows, at which the BTM can be classified as positive with the antibody ELISA used [8, 9].

The time from the introduction of a pathogen into the country, until it is detected by the surveillance activities, can be defined as “high risk period” (HRP) [10], or “timeliness” [7]. In this study we use the former term, to emphasize the fact that the longer the time required for detection the higher is the risk the pathogen is spread from the first case(s) herd(s) to other Danish herds.

The probability of detecting a pathogen in a given time period has been called “temporal sensitivity” [11].

In two previous studies we estimated the detection time for BVD in Danish dairy herds [9] and the annual probability of BVD introduction into the Danish dairy population [12]. Here we considered Danish dairy herds and beef herds as two distinct populations in the country, as in Foddai et al. [12], and we evaluated the BVD surveillance system for the Danish dairy population (on national level).

Hence, the aims of the present study where (i) to evaluate the temporal sensitivity of the Danish BVD surveillance system (TemSSe) with fixed HRPs, according to different routes of BVDV introduction into Danish dairy herds (e.g. import of PI or TI animals), (ii) to investigate surveillance optimization, by taking into account the risk of BVDV introduction to the country, the herd size, and the antibody ELISA used on BTM; and (iii) to estimate (at country level) the confidence in low herd prevalence (PLow) and in complete freedom from BVD (PFree).

## Methods

**Fig. 1:**
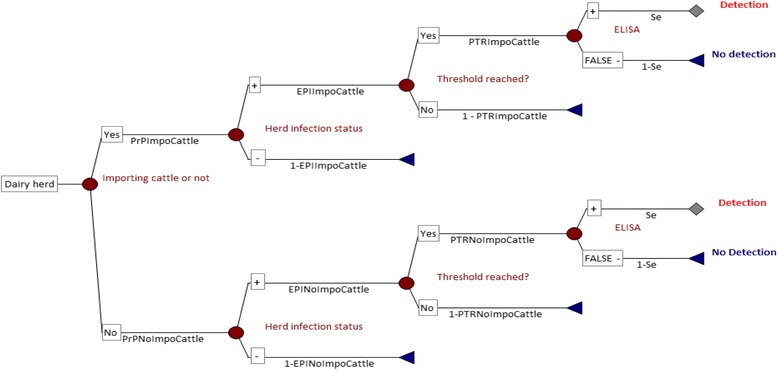
Stochastic scenario tree representing the BVD surveillance system for Danish dairy herds. PrP_ImpoCattle_ and PrP_NoImpoCattle_ = proportion of dairy herds within the ImpoCattle and NoImpoCattle category, respectively. EPI_ImpoCattle_ and EPI_NoImpoCattle_ = effective probability of infection for the ImpoCattle and NoImpoCattle category. PTR_ImpoCattle_ and PTR_NoImpoCattle_ = probability that the threshold prevalence is reached within the milking paddock at 90 or 365 days from BVDV introduction within herds of the ImpoCattle and NoImpoCattle category. Se = Expected sensitivity of the antibody ELISA used (Danish blocking ELISA or SVANOVIR) on BTM, when the threshold prevalence of seropositive milking cows is reached

To estimate the TemSSe of the Danish surveillance system, we developed a stochastic scenario tree model (Section Stochastic scenario tree methodology used to estimate the TemSSe-Input parameters used in the stochastic scenario tree, Fig. 1) [13–15]. Inputs for the model were obtained by data analysis (Section Data analysis), risk assessment (Section Risk assessment per herd category) and modelling of within-herd BVD dynamics (Sections Modelling the within-herd BVD dynamics to estimate the PTR values-Threshold prevalence and sensitivity of the ELISAs used for BTM testing). Thereafter, the estimated TemSSe was used to calculate the negative predictive value (NPV) of the surveillance system (Section Negative predictive value of the surveillance system) [13], which represented the confidence in low herd prevalence (PLow) or in complete freedom from BVD (PFree) according to the design prevalence used. Finally, a sensitivity analysis (Section Sensitivity analyses) was carried out to investigate the impact of each input parameter on the output.

### Stochastic scenario tree methodology used to estimate the TemSSe

The sensitivity of national veterinary surveillance systems (SSe) can be evaluated through use of stochastic scenario tree models [13–15]. Information from different surveillance sources can be combined into an overall SSe estimate, taking into account (I) the prevalence of infected herds in a country (between-herds design prevalence, P_H_), (II) the prevalence of infected animals within a herd (within-herd design prevalence, P_U_), (III) the relative risk (RR) of infection in the population´s strata, and (IV) the sensitivity (Se) of the diagnostic test used [13].

Thus the SSe represents the probability that a population, infected with the assumed P_H_ and P_U_, is correctly classified by the surveillance system [13–15]. Furthermore, in the methodology by Martin et al. [13] it is usually assumed that the specificity of the surveillance system is 100 %. Thus, if a positive sample is found, further confirmatory testing is made in the herd to avoid false positive results. In this study, we made the same assumption.

We developed a stochastic scenario tree (Fig. 1) in an Excel spreadsheet (Microsoft Office Excel 2007) using the software @Risk 6 (Palisade Corporation). The model was run using 10,000 iterations and Latin Hypercube sampling.

The P_H_ was set at 0.2 % (≈ 8 infected herds out of 4109 Danish dairy herds present in October 2010), or as 0.02 %, indicating that we aimed at detecting the first infected herd. The 0.2 % P_H_ is similar to the limit set by the World Animal Health Organization (OIE) to substantiate officially free status for infectious bovine rhinotracheitis (IBR) [16], which can be considered a disease similar to BVD, in terms of transmission routes. In fact, IBR is also caused by a virus, which can be transmitted between cattle herds and countries by moved animals, contaminated semen, embryos, and fomites [8, 17, 18]. Furthermore, these very low design prevalences used in this study give conservative TemSSe estimates, as the lower the disease prevalence between herds the more difficult it is to detect the disease.

P_U_ was here defined as the threshold prevalence of antibody positive milking cows, at which the BTM was classified positive by the ELISA used (see Section Threshold prevalence and sensitivity of the ELISAs used for BTM testing). With “fast” spreading pathogens, it can be assumed that P_U_ is quickly reached. In contrast, BVDV can be considered, in some cases, as a “slowly” spreading virus, especially if it is introduced into large herds through TI animals [9, 19]. Therefore, a long time could elapse before P_U_ is reached within a herd and before such a herd is detected by the surveillance system (long high risk period or HRP). For that reason, we estimated the temporal sensitivity of the surveillance system (TemSSe) for two different ELISAs, with a HRP of 90 or 365 days. Those two time intervals lead to TemSSe testing quarterly (current system) or testing one year after BVDV introduction into the Danish dairy population (as a worst case scenario), respectively. The effect of introducing one PI calf or one TI milking cow into naïve Danish dairy herd(s), on the final TemSSe was also investigated.

Moreover, dairy herds were divided into two levels of risk: herds with import of live cattle (ImpoCattle category) or without (NoImpoCattle category) (Fig. 1), based on a previous study [12].

### Input parameters used in the stochastic scenario tree

We used data from the last trimester of 2010, when approximately 4109 herds (N) delivered milk and were tested in the BTM. Hence, we considered a single surveillance component, which includes the whole population of Danish dairy herds.

The steps needed for a BVD positive dairy herd, to give a positive BTM value are represented with nodes in the scenario tree in Fig. 1. The first node divided the population of tested dairy herds into the two risk categories: NoImpoCattle and ImpoCattle. Thus, the proportion of dairy herds in each risk category was represented by PrP_NoImpoCattle_ and PrP_ImpoCattle_, respectively (Fig. 1).

The probability that a herd was infected with BVD was represented by the effective probability of infection in the category (EPI_NoImpoCattle_ and EPI_ImpoCattle_) (Fig. 1). The EPI_j_ for each herd category “j” was obtained by multiplying the between-herds design prevalence (P_H_) with the adjusted relative risk (ARRj) of infection [13, 14]. The relative risk estimates of each category (RR_NoImpoCattle_ and RR_ImpoCattle_) were adjusted to maintain the specified relativity (weight) and yet average to one over the whole population [15].

The NoImpoCattle category was used as risk reference category with RR_NoImpoCattle_ = 1, while for the ImpoCattle category we set RR_ImpoCattle_ = R_ImpoCattle_/R_NoImpoCattle_. Where, the numerator and denominator represented the risk of BVD introduction for the ImpoCattle and NoImpocattle category, respectively (Sections Risk assessment per herd category and Risk estimates per herd category).

Thereafter, the ARR for the NoImpoCattle category was estimated as:

1 $$ \mathrm{A}\mathrm{R}{\mathrm{R}}_{\mathrm{NoImpoCattle}}=\frac{1}{\left(\mathrm{PrPNoImpoCattle}+\mathrm{RRImpoCattle}\times \mathrm{PrPImpoCattle}\right)} $$

While, the ARR for the ImpoCattle category was:

2 $$ \mathrm{A}\mathrm{R}{\mathrm{R}}_{\mathrm{ImpoCattle}}=\mathrm{A}\mathrm{R}{\mathrm{R}}_{\mathrm{NoImpoCattle}}\times \mathrm{R}{\mathrm{R}}_{\mathrm{ImpoCattle}} $$

Furthermore, the component unit sensitivity was calculated as the weighted sum of the probabilities with a positive outcome at the end of each limb of the stochastic scenario tree (Fig. 1). Therefore, at the assumed P_H_, the overall probability (TemSSe) that at least one BTM positive herd was detected in the BTM surveillance system was:

3 $$ \mathrm{TemSSe}=1\hbox{-} {\left[1\hbox{-}\ \left( \Pr {\mathrm{P}}_{{}_{{}_{{}_{{}^{\mathrm{N}\mathrm{oImpoCattle}}}}}}\times \kern0.5em \mathrm{E}\mathrm{P}{\mathrm{I}}_{{}_{{}_{{}^{{}_{\mathrm{N}\mathrm{oImpoCattle}}}}}}\mathrm{x}\kern0.5em \mathrm{P}\mathrm{T}{\mathrm{R}}_{{}_{{}_{{}_{{}^{\mathrm{N}\mathrm{oImpoCattle}}}}}}\times \kern0.5em \mathrm{S}\mathrm{e} + \Pr {\mathrm{P}}_{{}_{{}^{{}_{{}_{{}_{\mathrm{I}\mathrm{mpoCattle}}}}}}}\times {\kern0.5em }_{\mathrm{EP}{\mathrm{I}}_{{}_{{}^{{}_{\mathrm{I}\mathrm{mpoCattle}}}}}}\times \kern0.5em \mathrm{P}\mathrm{T}{\mathrm{R}}_{{}_{\mathrm{I}\mathrm{mpoCattle}}}\times \kern0.5em \mathrm{S}\mathrm{e}\right)\right]}^{\mathrm{N}} $$

Where, PTR_NoImpoCattle_ and PTR_ImpoCattle_ represented the probability that the threshold prevalence of seropositive cows (P_U_) was already reached within the milking group at the day of BTM testing (Sections Modelling the within-herd BVD dynamics to estimate the PTR values-Threshold prevalence and sensitivity of the ELISAs used for BTM testing), in NoImpoCattle or ImpoCattle herds, respectively.

The Se was the sensitivity of the test used on the BTM sample. We assumed that the test´s Se could be achieved, when the threshold prevalence was reached. A similar approach was used by Graat et al. [8] for IBR. When the threshold prevalence was not reached, we assumed that the BTM of an infected dairy herd was classified as negative.

### Data analysis

Descriptive data analyses were carried out using the freeware R (R Development Core Team, 2012). Data from 2010, on herd size, milk deliverance, imports/exports of live cattle, imports of semen and embryos, were obtained from the Danish Cattle Federation for all Danish dairy cattle herds.

Moreover, for both herd categories, we estimated the percentage of imported goods from countries without or with endemic BVD (Table 1). We made this distinction on the BVD status of the sending countries, because it could affect the risk of introducing BVDV into the Danish cattle population [12].

**Table 1:** Results of data analysis for herds with import of cattle (ImpoCattle) and without (NoImpoCattle)

Herd Category	N	Imported cattle	Imported doses of semen	Imported embryos	Truck visits	Hoof trimmer visits^c^	PrP	EPI_j_^d^
ImpoCattle	8	246 (10.6 %)	3776 (92.9 %)	5 (100 %)	5606 * 0.8 %^b^ = 45	A*B* 0.2 %	0.2 %	0.025 %
NoImpoCattle	4101	0	301020 (46.9 %)	272 (99.3 %)	5606 * 99.2 %^b^ = 5561	A*B* 99.8 %	99.8 %	0.024 %
Total	4109	246^a^	304796^a^	277 ^a^	5606^a^		100 %	

*N* number of dairy herds delivering milk and tested in the fourth trimester of 2010 within each category, *PrP* proportion of herds within each category, *EPIj* effective probability of infection for each category “j”. Between brackets is the percentage of cattle, doses of semen and embryos imported from countries where BVD is endemic. We assumed that trucks and hoof trimmers went only to countries with endemic BVD status [12]

^a,^ From Foddai et al. [12]

^b,^ According to Foddai et al. [12], in total 5606 truck visits at risk occur in Danish dairy herds during a one-year period. The estimated percentage of exports from the ImpoCattle and the NoImpoCattle category was 0.8 % and 99.2 %, respectively (Danish data 2010). We assumed that the number of trucks visits at risk in each category was proportional to the exports occurred from the category

^c^ The number of hoof trimmers visiting cattle herds abroad (A) during a one-year period was a Pert distribution (5, 7, 18), while the number of times each hoof trimmer crosses the border (B) was Pert (1, 8, 30) (see Table 8 in [12]). The annual number of hoof trimmer visits, which could lead to BVDV introduction into each category, was assumed proportional to the herds present in the category and was estimated by: A * B * PrP_ImpoCattle_ and A * B * PrP_NoImpoCattle_

^d^The EPIj reported in the table was calculated using P_H_ 0.02 %. When we used P_H_ 0.2 % the EPIj was 0.20 for the ImpoCattle category and 0.19 % for the NoImpoCattle

### Risk assessment per herd category

The annual risk of BVDV introduction in each risk category (R_ImpoCattle_ and R_NoImpoCattle_) was calculated by use of a stochastic model, which was previously developed by Foddai et al. [12]. Results of the data analysis (Table 1) were fed into such a model.

For the NoImpoCattle category, we included the following sources of infection: import of semen and embryos, visits by contaminated trucks used abroad, and visits by hoof trimmers practicing in cattle herds in Denmark and in other countries. For the ImpoCattle category, we included the same BVDV introduction routes plus imports of live cattle.

Information on truck visits and hoof trimmers was based on the previous study [12]. The overall annual number of truck visits, which could lead to introduction of BVDV into Danish dairy herds, was previously estimated to 5606 [12]. For each of the two herd categories, we assumed that the number of truck visits at risk occurring in a year (or in a trimester) was proportional to the number of exports from the category (Table 1).

The annual number of hoof trimmer visits, which could lead to BVDV introduction into each category, was also estimated (Table 1) and was assumed to be proportional to the number of herds present in each category.

For other variables (e.g. the within-herd prevalence abroad, the probability of removing BVDV from contaminated trucks by disinfection, etc.), we used the same assumptions and input values described in Foddai et al. [12].

Hence, the risk of BVDV introduction in the ImpoCattle category (R_ImpoCattle_) was estimated using five published stochastic scenario trees [12], which represented: live-animal imports (PAnim), semen imports (PSem), embryo imports (PEmb), truck visits (PTruck) and hoof trimmers practicing across borders (PTrim). Thus, information from the five trees was combined in Eq. 4, as:

4 $$ {\mathrm{R}}_{\mathrm{ImpoCattle}}=1\hbox{-} \left[\left(1\hbox{-} \mathrm{PAnim}\right)\times \left(1\hbox{-} \mathrm{PSem}\right)\times \left(1\hbox{-} \mathrm{PEmb}\right)\times \left(1\hbox{-} \mathrm{PTruck}\right)\times \left(1\hbox{-} \mathrm{PTrim}\right)\right] $$

The risk (R_NoImpoCattle_) of BVDV introduction into the NoImpoCattle category was estimated using the same formula (Eq. 4) without the term (1-PAnim).

### Modelling the within-herd BVD dynamics to estimate the PTR values

The prevalence of antibody positive milking cows was assumed to increase over time [9]. The probability (PTR) of reaching the threshold prevalence (or P_U_ within the milking group) needed for BVD detection by BTM testing within a fixed HRP, was estimated using a stochastic simulation model; which was previously developed to simulate within-herd BVD dynamics and to estimate the detection time in Danish dairy herds, according to the ELISA used on BTM [9].

The PTR is affected by the route of BVDV introduction to the herd (PI or TI animal), the herd size, the threshold prevalence of the antibody ELISA used, and the time elapsed between BVDV introduction and day of testing (HRP). Thus, the PTR was calculated as the number of model iterations out of 500, where the threshold prevalence was reached, at 90 or 365 days after the introduction of one PI calf or one TI milking cow, into a naïve Danish dairy herd. A total of 500 iterations appeared sufficient to simulate the within-herd disease dynamics in the study by Foddai et al. [9], where the model was validated using data from a recently infected Danish dairy herd.

The estimation of PTR was carried out for the minimum, most common, and maximum herd size within each herd category. Thereafter, the PTR values were used in the stochastic scenario tree (Fig. 1, Eq. 3) in a Pert distribution (PTR_NoImpoCattle_ and PTR_ImpoCattle_) to represent the variability, between herds of different sizes within each risk category.

In the NoImpoCattle category, the herd size was minimum 1, median 123 and maximum 1185 cows (source: Danish data, 2010). For the smallest herd we did not run the simulation model, because we considered the threshold prevalence as reached soon, after the first infectious animal was introduced into the herd. Therefore, for this herd size we used PTR 100 %. In the ImpoCattle category, the herd size was minimum 24, median 180 and maximum 1070 cows.

Furthermore, in Foddai et al. [9], it was assumed that on average Danish dairy herds have around 150 cows, 115 heifers and 8 calves. Here, we used the same herd structure with a similar ratio of heifers/cows and calves/cows (at day one in the model) (Table 2). All other parameters were kept unchanged from Foddai et al. [9], e.g. we used the same values for: the BVDV transmission rates within a cattle herd (βs), the inter-calving period, the probability of abortion, the culling rates, the dry period, the age at first calving, and the lactation length per individual cow (Table 2).

**Table 2:** Herd parameters used in the simulation model by Foddai et al. [9]

Parameter	Value
Small herds (cows, heifers, calves)	ImpoCattle (24, 18, 2)^a^, NoImpoCattle (1, 0, 0)^a^
Medium herds (cows, heifers, calves)	ImpoCattle (180, 138, 10)^a^, NoImpoCattle (123, 94, 6)^a^
Large herds (cows, heifers, calves)	ImpoCattle (1070, 820, 66)^a^, NoImpoCattle (1185, 908, 64)^a^
Culling rate per year for cows	Pert distribution (min = 32 %, mode = 38 %, max = 43 %)^b^
Culling rate per year for heifers	Pert (4, 7, 12 %)^b^
Culling rate per year for calves	Pert (5, 12, 17 %)^b^
Parity distribution (1^st^, 2^nd^, 3^rd^ and 4^th^)	(31, 27, 22, and 20 %)^b^
Percentage of dry cows	Pert (12, 17, 20 %)^b^
Age in the heifers group (in days)	(700; 768; 870)^b^
Days of inter-calving per cow between parity 1 and 2	(365, 399, 451)^b^
Days of lactation per cow between parity 1 and 2	(305, 339, 391)^b^
Days of inter-calving per cow after parity 2	(370, 391, 456)^b^
Days of lactation per cow after parity 2	(310, 331, 396)^b^

^a^ The median number of cows was set according to Danish data (2010), for herds with imports of cattle (ImpoCattle) and without (NoImpoCattle). According to a previous study [9], usually, Danish dairy herds have around 150 cows. In that case the number of heifers (animals with age between 61 and approximately 900 days) and calves (age up to 60 days) present in the herd corresponded to 76.7 % (115/150) and 5.4 % (8/150) of the number of cows. We used similar (approximated) heifers/cows and calves/cows ratios in the herd sizes of the two risk categories

^b^ Unchanged from Foddai et al. [9]

### Threshold prevalence and sensitivity of the ELISAs used for BTM testing

In a previous study [9], the SVANOVIR^®^BVDV-Ab ELISA (Svanova Boehringer Ingelheim, Uppsala, Sweden) [20–23] was shown to detect BVD by BTM testing, significantly earlier than the Danish blocking ELISA [24, 25], which has been traditionally used in Denmark. As a part of this study, we therefore wanted to evaluate the surveillance system with the two different ELISAs, both used to test BTM samples for antibodies against BVDV.

The threshold prevalence has been estimated at 50 % for the Danish blocking ELISA [1] and at 6 % for the SVANOVIR ELISA [23]. Those thresholds were used in the stochastic simulation model by Foddai et al. [9], to estimate the PTR for each herd size (Table 3).

**Table 3:** PTR values in herds of different size within each herd category (ImpoCattle or NoImpoCattle)

			PTR with 1 PI		PTR with 1 TI cow	
Herd category	Test	Herd size (in cows)	HRP = 90 days	HRP = 365 days	HRP = 90 days	HRP = 365 days
ImpoCattle	blocking ELISA	24	175 (35.0)	343 (68.6)	0 (0.0)	23 (4.6)
		180	0 (0.0)	206 (41.2)	0 (0.0)	2 (0.4)
		1070	0 (0.0)	14 (2.8)	0 (0.0)	0 (0.0)
	SVANOVIR	24	468 (93.6)	486 (97.2)	165 (33.0)	308 (61.6)
		180	240 (48.0)	376 (75.2)	0 (0.0)	38 (7.6)
		1070	3 (0.6)	281 (56.2)	0 (0.0)	9 (1.8)
NoImpoCattle	blocking ELISA	1	500 (100.0)	500 (100.0)	500 (100.0)	500 (100.0)
		123	0 (0.0)	248 (49.6)	0 (0.0)	3 (0.6)
		1185	0 (0.0)	8 (1.6)	0 (0.0)	0 (0.0)
	SVANOVIR	1	500 (100.0)	500 (100.0)	500 (100.0)	500 (100.0)
		123	314 (62.8)	397 (79.4)	2 (0.4)	47 (9.4)
		1185	1 (0.2)	278 (55.6)	0 (0.0)	12 (2.4)

*PTR* Iterations out of 500 (in %), where the threshold prevalence of antibody positive milking cows was reached in a herd, using the stochastic simulation model by Foddai et al. [9] and according to test used (blocking ELISA vs. SVANOVIR) sampling day (HRP of 90 or 365 days), and BVDV introduction route (PI calf or TI cow)

With a cut-off blocking % (bl%) of 50, the sensitivity (Se) of the Danish blocking ELISA on BTM is 100 % [26], while for the SVANOVIR ELISA, the Se has been estimated between 93.4 % and 99.6 % [27]. In the latter case, a Uniform distribution ranging between those two extremes was used in the Se input (Fig. 1, Eq. 3).

### Negative predictive value of the surveillance system

The scenario tree methodology [13–15] is usually used to substantiate freedom from a disease or from a pathogen (PFree) at country/area level. For that purpose, the negative predictive value (NPV or PFree) of the surveillance system is estimated to represent the confidence that a country, classified as free from a pathogen by the system, is truly free. In that case, the design prevalence (P_H_ and P_U_) represents a hypothetical level of infection in the country. If a single positive unit is found, the country would lose the “free status”.

In our study, we estimated the NPV of the surveillance system to show the PFree, but we also estimated the confidence (PLow) that the prevalence of infected herds is below the P_H_. In the latter case, we did not exclude that in reality few positive herds could be present in the country.

Thus, to estimate PLow we used the TemSSe based on P_H_ = 0.2 %, while to estimate PFree the TemSSe was obtained using P_H_ = 0.02 %. The NPV of the surveillance system was:

5 $$ \mathrm{PLow}=\frac{\left(1\hbox{-} \mathrm{PriorPInf}\right)}{\left(1\hbox{-} \mathrm{PriorPInf}\right)+\left(\mathrm{PriorPInf}\kern0.5em \times \kern0.5em \left(1\hbox{-} \mathrm{TemSSe}\right)\right)} $$

Where PLow (or PFree) is the confidence that the prevalence of infected herds was below the assumed design prevalence 0.2 % (or 0.02 %) at the beginning of the surveillance period, since no BVD cases have been detected with the investigated HRP.

PriorPInf is the probability that the country was “infected” with the assumed design prevalence, at the beginning of the surveillance period. This input was set to 50 %, which corresponds to a conservative uninformed prior [13].

Moreover, the PriorPInf was adjusted (PriorPInf_Adj_) (Eq. 6), by taking into account the probability of BVDV introduction from abroad (PIntro):

6 $$ \mathrm{PriorPInfAdj}=\mathrm{PriorPInf}+\mathrm{PIntro} - \left(\mathrm{PriorPInf}\times \mathrm{PIntro}\right) $$

The annual median PIntro for Danish dairy herds has been estimated as 10.7 % (90 % prediction interval: 1.7 %; 36.6 %) [12]. This PIntro was used in Eq. 6 as a Pert distribution, for the scenarios with 365 days. With scenarios of 90 days the PIntro was divided by 4.

Thus, PLow and PFree were estimated for each infection scenario (introducing a PI or a TI animal into 1 or 8 dairy herds), HRP (90 or 365 days) and ELISA used.

### Sensitivity analyses

The importance of each input parameter was investigated by using the regression mapped values in @Risk and the amount of change in the output due to a plus 1 standard deviation for each input was estimated.

The reference scenario was defined as: all dairy herds tested with the Danish blocking ELISA (current system) one year after introduction of a PI calf into a single dairy herd (P_H_ = 0.02 %), since PIs are the main source of BVDV spread between and within herds [28].

## Results

### Output of data analysis

The imports of live animals, doses of semen, embryos, and the visits of trucks and hoof trimmers are shown per herd category in Table 1. The percentage of goods imported from endemic countries is also shown for each category.

In 2010 cattle were imported to eight dairy herds, which represented the ImpoCattle category. On the other hand, the annual quantity of imported semen and embryos, as well as the estimated numbers of trucks and hoof trimmer visits were remarkably higher for the NoImpoCattle category than for the ImpoCattle category.

### Risk estimates per herd category

The median annual risk of BVDV introduction in the NoImpoCattle category (R_NoImpoCattle_) was 4.8 % (90 % prediction interval: 0.7 %, 21.8 %), while in the ImpoCattle category (R_ImpoCattle_) it was 5.1 % (0.7 %, 22.4 %).

Based on those findings, the relative risk of BVDV introduction in the ImpoCattle category (RR_ImpoCattle_) was calculated as the ratio between the two median risk estimates (and between their respective 90 % prediction intervals). Thus, the RR_ImpoCattle_ was simulated from a Pert distribution with minimum 1, mode 1.03 and maximum 1.07, to calculate the ARR_NoImpoCattle_ (Eq. 1) and the ARR_ImpoCattle_ (Eq. 2).

### PTR values according to infection scenario, herd size, HRP and ELISA

The PTR values were higher: i) in small herds than in large herds, ii) for BVDV introductions through one PI calf than through one TI milking cow, and iii) with HRP of 365 days than with HRP of 90 days. Moreover, the SVANOVIR ELISA had higher PTR values than the Danish blocking ELISA (Table 3).

In the ImpoCattle category, the PTR ranged from 0 %, e.g. when a PI calf or a TI cow was introduced into the largest herd (with 1070 cows), and the BTM was tested 90 days later with the blocking ELISA; to 97.2 % when one PI calf was introduced into the smallest herd (24 cows), and the BTM was tested one year later with the SVANOVIR (Table 3).

In the NoImpoCattle category the PTR ranged from 0 %, e.g. when a PI calf or a TI cow was introduced into the largest herd (1185 cows), and the BTM was tested 90 days later with the blocking ELISA; to 100 % when one infectious animal was introduced into a herd of a single cow (Table 3).

### Temporal sensitivity and negative predictive value of the surveillance system with P_H_ = 0.2 %

If BVDV was introduced into at least 8 dairy herds (P_H_ = 0.2 %), with the Danish blocking ELISA (current surveillance system), the median TemSSe ranged from 64.4 % to 98.2 %. The related median PLow estimates were 72.5 % and 97.6 %, respectively (Table 4).

**Table 4:** Temporal surveillance sensitivity with related confidence in low herd prevalence and in freedom from disease

P_H_ = 0.2 %	1 PI introduced		1 TI introduced	
ELISA_HRP	TemSSe	PLow	TemSSe	PLow
blocking_90	64.5 (7.9; 97.3)	72.5 (50.4; 97.2)	64.4 (7.8; 97.3)	72.5 (50.4; 97.2)
SVANOVIR_90	99.0 (86.9; 99.9)	98.9 (87.7; 99.9)	64.0 (8.0; 97.0)	72.1 (50.4; 96.9)
blocking_365	98.2 (79.2; 99.8)	97.6 (78.5; 99.8)	65.6 (8.5; 97.4)	68.9 (44.9; 96.7)
SVANOVIR_365	99.8 (99.3; 99.9)	99.7 (99.1; 99.9)	78.9 (30.5; 98.3)	78.3 (52.0; 97.8)
P_H_ = 0.02 %	1 PI introduced		1 TI introduced	
ELISA_HRP	TemSSe	PFree	TemSSe	PFree
blocking_90	12.1 (1.0; 36.2)	51.6 (48.2; 59.5)	12.1 (1.0; 36.2)	51.6 (48.2; 59.5)
SVANOVIR_90	43.7 (22.6; 56.9)	62.4 (54.6; 68.6)	12.0 (1.0; 35.5)	51.5 (48.2; 59.2)
blocking_365	39.3 (17.8; 55.4)	55.7 (46.3; 64.5)	12.5 (1.1; 36.6)	47.3 (40.0; 55.8)
SVANOVIR_365	53.5 (46.7; 59.2)	62.3 (55.5; 67.3)	17.7 (4.4; 40.0)	48.6 (41.0; 57.2)

*TemSSe* Median temporal surveillance system sensitivity, *PLow* Confidence in low herd prevalence, *PFree* Confidence in complete freedom from BVD. Estimates are reported as median % (90 % prediction intervals), according to ELISA used (blocking = Danish blocking ELISA, SVANOVIR = SVANOVIR ELISA), and HRP (high risk period of 90 or 365 days) after the introduction of 1 persistently infected (PI) calf or 1 transiently infected (TI) milking cow into 1 (between-herds design prevalence P_H_ = 0.02 %) or 8 (P_H_ = 0.2 %) Danish dairy herd(s)

Using the same P_H_, with the SVANOVIR ELISA, the median TemSSe ranged from 64.0 % to 99.8 %. The related median PLow estimates were 72.1 % and 99.7 %, respectively (Table 4).

Usually, the blocking ELISA gave lower TemSSe and PLow estimates than the SVANOVIR. For instance, when one PI calf was introduced into at least eight dairy herds and a HRP of 90 days was used, the median TemSSe and the PLow were 34.5 and 26.4 percentage points (respectively) higher for the SVANOVIR ELISA (Table 4).

Only in the scenario where one TI cow was introduced to the herds and a HRP of 90 days was used, the median TemSSe and the PLow were slightly higher (around +0.4 %) for the blocking ELISA. With a HRP of 365 days the opposite situation was observed, and the TemSSe and the PLow were remarkably higher for the SVANOVIR (+13.3 and +9.4 %, respectively) than for the blocking ELISA (Table 4).

### Temporal sensitivity and negative predictive value of the surveillance system with P_H_ = 0.02 %

If BVDV was introduced into a single dairy herd (P_H_ = 0.02 %), and all Danish dairy herds were tested with the blocking ELISA, the median TemSSe ranged from 12.1 % to 39.3 %. The related median PFree estimates were 51.6 % and 55.7 %, respectively (Table 4).

Using the same P_H_, with the SVANOVIR, the median TemSSe ranged from 12.0 % to 53.5 %, while the related PFree estimates were 51.5 % and 62.3 %, respectively (Table 4).

Also in this case, usually, the TemSSe and the related PFree were higher in the surveillance system based on the SVANOVIR ELISA. For instance, when one PI calf was introduced into a dairy herd and a HRP of 90 days was used, the median TemSSe and the PFree were 31.6 and 10.8 percentage points (respectively) higher for the SVANOVIR ELISA than for the blocking ELISA (Table 4).

### Output of sensitivity analysis

According to the sensitivity analysis, the input with the highest impact on the estimated TemSSe was the PTR distribution used in the NoImpoCattle category (PTR_NoImpoCattle_). When this input was increased with 1 standard deviation the TemSSe increased between 0 and 11.4 %. The second input in order of importance was the PTR distribution used in the ImpoCattle category (PTR_ImpoCattle_). In that case, the increase caused on the TemSSe ranged between 0 and 0.03 %. The other inputs caused a change lower than 0.03 %.

For the PFree, the most important input was still the PTR_NoImpoCattle_. Increasing such an input with one standard deviation caused an increase in the PFree between 0 and 4.5 %. The second most important input was the annual (overall) probability of BVDV introduction (PIntro) into the Danish dairy population. Increasing the PIntro of 1 standard deviation caused a decrease in the PFree between 0 and 3.1 %. All the other inputs caused a change lower than 0.006 %.

## Discussion

### A new approach – Including the high risk period in the stochastic scenario tree methodology

To evaluate the BVD surveillance system in Danish dairy herds, we followed the concepts from Martin et al. [13]. Additionally, we estimated the temporal surveillance system sensitivity (namely the TemSSe) and the related negative predictive value (Eq. 5), to substantiate the confidence (PFree) in complete freedom from BVD (< 1 infected herd), and the confidence (PLow) in low herd prevalence (< 8 infected herds).

The way we adapted the scenario tree model allowed us to estimate the TemSSe. Thurmond [11] suggested that the sensitivity for an assay should not be considered as constant, since it is affected by the different disease states. This means that the sensitivity of the test is affected by the time elapsed since a herd (or an animal) became infected. In our case, the transition state herd sensitivity of the ELISA used on BTM, was conditioned upon the immune status of the milking herd. That status varied in time according to HRP, test used, BVDV introduction route (with PI or TI animals) and herd size. Uncertainty, due to all these variables was included in our TemSSe estimates (Table 4), by using the PTR parameter (Table 3) in the scenario tree (Fig. 1), between the infection node “Herd infection status” and the detection node “ELISA”.

In this way, we could evaluate if the surveillance system can actually function as an early-warning system, or if optimization was needed, to increase the probability of detecting infected herds (TemSSe) within the aimed time period.

To our knowledge, this is the first study, where the impact of the HRP is included in the evaluation of a surveillance system using stochastic scenario trees. We believe that this approach should be considered, especially when early-warning surveillance systems are established for slowly spreading diseases, as is the case of BVD in large dairy herds after introduction of TI animals [9, 19].

### Temporal sensitivity and confidence in low herd prevalence (TemSSe and PLow)

Conclusions on surveillance sensitivity and disease status at national level should be related to a specific time period, when the pathogen could have been introduced into the country. Hence, in our case, the TemSSe and PLow/PFree should be related to the period when the BVDV could have been introduced into the Danish dairy herd(s).

Between December 2011 and December 2012, no dairy herds have been found positive in Denmark. Therefore, we can assume that very few (≤ 8) or no dairy herds were infected in the country at the beginning of 2012 and became BTM positive. Currently, there is no regulated between-herds design prevalence to substantiate BVD status at country level [29]. Thus we considered P_H_ = 0.2 % as a reasonable cut-off level. Such design prevalence has been set up by the OIE to substantiate officially free status from IBR [16], which has been eradicated from Denmark [17].

If BVDV was introduced by a PI calf in at least 8 dairy herds, the median probability of detecting at least one of these herds after one year by BTM testing, would be > 95 % with both ELISAs. According to the European Food Safety Authority (EFSA) this could be considered as an acceptable level of confidence (e.g. when no value is indicated in the legislation) [30]. The confidence in low herd prevalence (PLow) would be high as well (Table 4). This means that if the aim of the surveillance system was to substantiate on annual basis that the prevalence of herds infected by at least one PI animal is <0.2 %, there is no need to replace the Danish blocking ELISA with the SVANOVIR ELISA.

On the other hand, if the objective of the surveillance system was to detect BVD by 90 days after introduction of a PI calf in 0.2 % dairy herds, then the SVANOVIR ELISA could be preferred, because only that test showed median TemSSe and PLow higher than 95 % (Table 4). PI animals are usually considered to be the major sources of BVDV spread [28], between and within cattle herds, and considering BVDV introductions by those animals could therefore be sufficient.

As we showed with the PTR values (Table 3), outbreaks due to TI cows can occur with low probabilities, and if the objective of the surveillance system is to detect BVD after introduction of a TI cow in 0.2 % dairy herds, the SVANOVIR ELISA could be used, although in that case, the median TemSSe and the PLow would be < 95 % (Table 4).

### Confidence in complete freedom from BVD (PFree)

With P_H_ = 0.02 % (corresponding to 1/4109 infected herds), the TemSSe and the related PFree were <95 %, with both ELISAs (Table 4).

Hence, if we apply those findings to the BTM testing made in the fourth trimester of 2010, with the Danish blocking ELISA, it can be concluded that the probability of detection and the confidence in complete freedom from BVD, one year after one single herd was infected by a PI calf were very low (Table 4).

However, if the SVANOVIR ELISA had been used, under the same infection and HRP scenarios, the TemSSe and the related PFree would have been higher than with the Danish blocking ELISA (Table 4).

### Impact of herd size, HRP and ELISA on the TemSSe and its related PLow/PFree

In this study, we found that, if one TI cow was introduced to the herd(s) and a HRP of 90 days was used, the TemSSe and the related PLow/PFree were similar or slightly higher for the blocking ELISA than for the SVANOVIR (Table 4). Under the same infection scenario, when a HRP of 365 days was used, the contrary was observed. This was due to the fact that, with an HRP of 90 days, detection occurred with both tests in the NoImpoCattle herd with one cow (where the PTR was 100 %). With the SVANOVIR, the PTR was >0 % also in the ImpoCattle herd with 24 cows and in the NoImpoCattle herd with 123 cows (Table 3). However, the Se of the SVANOVIR ELISA on BTM was assumed to be lower [27] than the Se of the Danish blocking ELISA [26].

Thus, when 1) the herd size is very small (e.g. <50 cows), 2) the threshold prevalence of the test used is low, and consequently 3) the PTR is around 100 %, the TemSSe would become more dependent on the Se of the test used on BTM, than on the PTR. Then there is less need to consider the threshold prevalence of the test used and the PTR parameter (the node “Threshold reached?” in Fig. 1) could be removed. In fact, in very small herds, even a high threshold prevalence of 50 % can be reached in a short HRP with very high probability (high PTR).

When the Danish BVD eradication program was launched in 1994, the average herd size was 42 cows [2], while currently it has increased to approximately 150 cows [9]. Hence, in the ´90s detection could occur with the Danish blocking ELISA, even testing quarterly and especially if a PI was introduced to the herd(s). In the current situation, this is more difficult, since the size of Danish dairy herds is continuously increasing [1] and the HRP (with the PTR value) have higher importance than in the past. Thus, the PTR needs to be used in the evaluation of the current surveillance system. With larger herds, tests that can detect a lower prevalence of seropositive animals, in a short HRP and with higher PTR should be preferred. This is the case of the SVANOVIR compared to the blocking ELISA. Using the former, a higher TemSSe would be achieved.

With those points in mind, it can also be noted that, once the threshold prevalence has been reached, increasing the BTM testing frequency would increase the costs related to the higher number of samples tested, but also the probability of detection (TemSSe). In contrast, using a higher BTM testing frequency, before the threshold prevalence is reached in the milking group, would be inefficient. In the latter case, changing the test (rather than increasing the testing frequency), could increase the TemSSe without increasing the costs of the surveillance system (if we assume that tests have similar commercial price from the manufacturer).

### Importance of disease epidemiology and infection scenario

In this study, we showed that the TemSSe was higher for HRP of 365 days than for HRP of 90. This was due to two main reasons: a) the longer the time an infectious animal is kept in the herd, the higher the probability that such an animal causes an outbreak with seroconversion of several milking cows, and b) within the first 90 days from BVDV introduction, no new PI calves could be born in the herd from recently infected cows (TI).

The first observation is also valid for other diseases, while the second is peculiar to the epidemiology of BVD. In fact, PI calves are born from PI cows or from susceptible cows, which become infected within the first four months of pregnancy [4, 5]. In the latter case, PI calves will be born in the herd at least 5 months after introduction of the first infectious animal (one PI calf or one TI cow in our infection scenarios), because the cattle pregnancy lasts around 280 days.

When PIs are present in a herd, the immunization of other herd mates occurs quicker than when only TI animals are present [9]. In fact in simulation studies, it is usually assumed that the within group transmission rate of TIs is approximately 17 times lower than in PIs, and that only the latter are able to spread BVDV between animals groups (e.g. from calves to milking cows) [9, 31, 32]. Thus, detection of BVD infected herds by BTM testing becomes more likely (higher PTR) in the presence of PI cattle, since the number of newly infected animals per unit of time is very high.

These observations are in accordance with our results. In most of the iterations where we introduced a TI and the within herd BVDV spread was simulated, the outbreak died out, before the threshold prevalence was reached. Thus, in those scenarios, the TemSSe was low (Table 4) due to the low PTR values (Table 3).

Therefore, the approach proposed in this study, allowed us to evaluate the Danish BVD surveillance system taking into account all the main epidemiological characteristics of the disease.

### Information from sensitivity analysis

In the sensitivity analysis, we confirmed that the PTR is an important parameter to consider, when the temporal sensitivity of the surveillance system is estimated.

Moreover, we showed how an increase in the probability of BVDV introduction into the Danish dairy population (PIntro) could cause a decrease in the PFree. Testing imported animals at the border, could reduce the PIntro and could increase the confidence in freedom (PFree), as previously argued for bovine tuberculosis [33].

### Limitations of the study

Our estimates (TemSSe, PLow and PFree) could be considered as conservative, since we assumed that herds became infected by introduction of one BVDV positive animal only (a PI or a TI). In reality, more infected animals could be introduced to one herd at the same time leading to higher values of PTR and TemSSe.

Moreover, the PTRs were estimated for three herd sizes within each herd category. A more precise modeling for all herd sizes would have required to run the simulation at least 1185 times, to determine the PTR for each dairy herd size (from 1 to 1185 cows). Because this was not feasible, to include uncertainty, we set the PTR values as Pert distributions within each risk category (Fig. 1).

In the model used for the risk assessment [12], we did not include veterinarians visiting infected cattle herds abroad. This was based on interviews with farmers and vets, who stated that the veterinary equipment and medicines used in herds outside Denmark were not used in Danish herds [12].

Finally, in line with Foddai et al. [9] we assumed that detection by BTM testing could occur when a fixed threshold prevalence of antibody positive milking cows was reached within a herd, and that, these cows had similar milk production and antibody levels in milk. In reality, this is not always the case, and thus, we used a simplification. Further studies could investigate how the sensitivity of the ELISA used on BTM samples changes per day (after the introduction of the infectious animal(s)), according to prevalence of seroconverted milking cows, their individual milk production and antibody titer.

## Conclusions

Using the SVANOVIR ELISA on BTM, would increase the temporal sensitivity and the related confidence in BVD freedom (and in low herd prevalence), compared to the current situation, where the Danish blocking ELISA is used. Those results could be considered to optimize the BVD surveillance system in Danish dairy herds and to substantiate freedom from disease in the Danish dairy population. Moreover, in this study, we showed a novel idea on how to include the effect of the high risk period within the stochastic scenario tree methodology. By using this approach surveillance systems of different animal diseases could be evaluated and optimized as early warning systems.

## Abbreviations

ARR_ImpoCattle_, adjusted relative risk of infection for the ImpoCattle category; ARRj, adjusted relative risk of infection for the risk category “j”; ARR_NoImpoCattle_, adjusted relative risk of infection for the NoImpoCattle category; BTM, bulk tank milk; BVD, bovine viral diarrhea; BVDV, bovine viral diarrhea virus; EFSA, European food safety authority; ELISA, enzyme-linked immunosorbent assay; EPI_ImpoCattle_, effective probability of infection for the ImpoCattle category; EPIj, effective probability of infection for the category “j”; EPI_NoImpoCattle_, effective probability of infection for the NoImpoCattle category; HRP, high risk period; IBR, infectious bovine rhinotracheitis; ImpoCattle, dairy herds which import live cattle; NoImpoCattle, dairy herds which do not import live cattle; NPV, negative predictive value of the surveillance system; PAnim, annual risk of BVDV introduction due to import of live cattle (estimated only for the ImpoCattle category, Eq. 4); PEmb, annual risk of BVDV introduction due to import of embryos (estimated for each category); PFree, confidence in complete freedom from BVD (P_H_ < 0.02 % or <1/4109 infected herds); P_H_, between-herds design prevalence; PI, persistently infected cattle; PIntro, probability of BVDV introduction into the Danish dairy population; PLow, confidence in low herd prevalence (P_H_ < 0.2 % or < 8/4109 herds); PriorPInf, prior probability that the country is infected at the assumed P_H_ at the beginning of the surveillance period; PriorPinf_Adj_, prior probability that the country is infected at the assumed P_H_ at the beginning of the surveillance period adjusted for the PIntro; PrP_ImpoCattle_, proportion of dairy herds which import live cattle; PrP_NoImpoCattle_, proportion of dairy herds which do not import live cattle; PSem, annual risk of BVDV introduction due to import of semen (estimated for each category); PTrim, annual risk of BVDV introduction due to hoof trimmers practicing abroad (estimated for each category); PTR_ImpoCattle_, probability that the threshold prevalence is reached within the ImpoCattle herds on the day of testing; PTR_NoImpoCattle_, probability that the threshold prevalence is reached within the NoImpoCattle herds on the day of testing; PTruck, annual risk of BVDV introduction due to trucks used abroad (estimated for each category); P_U_, within-herd threshold/design prevalence of antibody positive milking cows; R_ImpoCattle_, risk of BVDV introduction for the ImpoCattle category; R_NoImpoCattle_, risk of BVDV introduction for the NoImpoCattle category; RR_ImpoCattle_, risk of BVDV introduction for the ImpoCattle category relative to the R_NoImpoCattle_; RR_NoImpoCattle_, relative risk of BVDV introduction for the NoImpoCattle category (equal to 1); Se, test sensitivity; SSe, surveillance system sensitivity; TemSSe, temporal surveillance system sensitivity; TI, transiently infected cattle
